# The Nature and Treatment of Deafness, and Diseases of the Ear, &c.

**Published:** 1849-01

**Authors:** 


### Part 2.—Reviews and Notices of New Works.

### ARTICLE I.

*The Nature and Treatment of Deafness, and Diseases of the Ear, and the Treatment of the Deaf and Dumb.* By WILLIAM DUFTON, M. R. C. S. pp. 120 8vo. Lea & Blanchard: Philadelphia, 1848. (From the publishers, and for sale by J. Keene & Bro., Chicago.)

In consequence of carelessness and ignorance, in reference to a proper diagnosis and treatment of the diseases of the ear, the public pays but little regard to the character of the employee in their management, and our author very correctly says, in his preface:—

"In having recourse to the empiric in cases of deafness, the public has not been so much to blame as the medical profession, for, in general, a superficial examination of the external ear was all that the surgeon thought necessary to make, and the remedies he prescribed seldom gave any relief." This is equally true, when applied to this country, as it is in Great Britain: and our enterprising publishers deserve the thanks of the profession for furnishing them with this little volume on a subject that we may safely say has received far less attention than its importance demanded, and in reference to which but little has been written. The great number of our population who are deaf and dumb, most of whom lose the sense of hearing in consequence of diseases of the ear, point at once to the importance of the subject, and show that an improvement in our knowledge of these diseases, and their treatment, is loudly called for.

Agreeably to the census of 1840, there were in the United States 7659 deaf and dumb persons; being about 1 to every 2000 of the population.

Although the eye is more exposed and more subject to disease, and although many diseases of the eye are destructive of the sight, still the proportion of the blind is considerably less.

In this comparison, it will be observed that only the deaf and dumb are considered, to which we may add a large number for those who have lost the sense of hearing at an age when they had acquired the power of speaking.

This is, probably, to a great extent, the result of an opinion which our author speaks of in his preface as obtaining to a considerable extent in the medical profession at one time, and which we apprehend is not entirely obsolete in this country yet, which is, "that diseases of the ear, producing deafness, could seldom or never be cured;" to which may be added such an entire want of knowledge on the subject, that few would be much better off without than with the influence of this opinion, while the eye has generally been studied thoroughly in its physiology, pathology, and therapeutics.

In view of this state of the case, and to call the attention of our professional brethren to the subject, we proceed to give a brief outline of the little work before us.

It is divided into four chapters. The first treats of "acute and chronic inflammations of the ear." The second of "diseases of the ear not purely inflammatory." The third of "nervous diseases of the ear;" and the last "on the treatment of the deaf and dumb."

We pass by the section devoted to the consideration of inflammations of the external ear, as they present no features peculiar or different from like conditions of other parts, and are treated upon the general principles by which other local inflammations are combatted. However, when the integuments lining the meatus auditorius externus are inflamed, the diseased action is quite prone to extend to the membrana tympani. Here we have a condition of danger to the integrity of the ear deserving attention.

The symptoms diagnostic of inflammation of the membrana tympani, are the appearances of the membrane, it becomes reddened, the dullness of hearing, a sensation resembling the the presence of a buzzing insect, with deep seated pain in the part, and general fever.

Mild cases may be relieved by fomentations to the external ear; in those that are more severe, leeches behind the ear, venesection, cathartics, and anodynes should be given; where these fail, our author recommends that a slight ptyalism be induced with calomel. The danger of introducing stimulating substances into the auditory canal, in inflamed conditions of the ear, as is generally practiced in all cases of ear-ache, is set forth, and the practice justly condemned by our author.

The unpleasant consequences of inflammation of the membrana tympani are thickening and ulceration of the membrane. As a local application, a solution of acetate of lead is highly recommended in the chronic form of the disease, especially where there are ulceration and suppuration. Our author insists very correctly upon the importance of frequent injections of warm water in chronic cases, for the purpose of removing the morbid secretions, that otherwise accumulate. The first part of the treatise is not so clear or concise as we could wish.

Section second treats of those affections from which deafness generally results. These are inflammations of the middle and internal ears.

Inflammation of the tympanum is treated as acute and chronic, under two heads. The acute is attended by decided febrile symptoms, which frequently are ushered in by rigors. Our author says:—

The first thing a patient complains of, in the midst of these symptoms, are acute pains deep in the ear, which are described as pricking, burning, tearing, boring, and dragging. These pains are usually confined to one ear, and are aggravated by every motion of surrounding parts, as in chewing, sneezing, coughing, stooping, and the like. The disease sometimes extends down the eustachian tube, over the pharynx and tonsils, and into the mastoid cells, and pains are felt over the temporal bone and towards the vertex or occiput. Sometimes the mastoid process is tender on pressure, and not unfrequently the vicinity of the whole ear is swollen; the eye of the affected side becomes increasingly sensitive to light, is suffused with tears, and reddened. As the disease progresses, the fever augments, the nights are perfectly restless, pain in the head of a most insupportable character coming on, and is attended with delirium, frequently of the most violent kind. The pulse is hard and frequent, thirst great, urine scanty and high colored, constipation, great heat of skin, and, not unfrequently, vomiting. These symptoms continue, and in the course of a few days the patient dies with all the indications of inflammation of the brain. But it frequently happens that, in the midst of the most violent symptoms, a purulent fluid suddenly escapes from the tympanum or out of the meatus, or sometimes an abscess forms, which bursts over the mastoid process, and in these cases the matter is frequently bloody, of a very offensive character, and contains fragments of bone, and even the small bones of the ear. Sometimes the discharge of matter takes place from the eustachian tube, but in most cases the tube is closed, at the commencement of the attack, by adhesive inflammation, or from tumefaction of its walls.

This affection is frequently fatal in consequence of the disease extending to within the cavity of the skull, and to the brain.

The absence of disease in the external ear, ascertained by examining the auditory canal, and absence of a discharge during the first stages of the disease will serve to distinguish cases of this kind from external otitis.

The treatment in the first stages must be actively antiphlogistic, anodyne, and mercurial, according to our author.

When suppuration has taken place, and the pus is confined by the closure of the eustachian tube, the case is extremely difficult to manage. If, upon examining the membrana tympani, it be convex, while there is tenderness over the mastoid bone, our author thinks its puncture would be justifiable. The great trouble would be in ascertaining the convexity.

The chronic form of the disease is illustrated by an interesting case, with an inspectio cadaveris, in which disease of the petrous bone caused an effusion of pus beneath the dura mater that had extended over a considerable portion of the skull before death. Two other cases are given, in which the patients were relieved by the use of injections through the eustachean tube by means of the catheter.

We next have internal catarrhal otitis, or inflammation of the mucuous membrane lining the tympanum. The closure of the eustachian tube in catarrh causes an accumulation within the tympanum that becomes, if the tube remains long closed, thickened by the absorption of the thinner portions, and the ear is partially exhausted. Opening the tube suddenly restores hearing with a cracking sound.

Obstructions of the eustachian tubes our author recommends to be removed by the catheter. And, inasmuch as the use of injections is advised, which have to be introduced to the middle ear by the chatheter through the tube, our author gives quite a brief account of the instrument and its use, from which we quote the following:—

*Catheterism.*—The diagnosis, however, of obstruction of the eustachian as well as of muculent accumulations in the middle ear, can only be accurately made out by the use of the catheter. As the application of catheterism to the eustachian tube is not generally practised, although it has been known for some time, it may be as well to say a few words here on the subject. This operation, it appears, was first performed on himself by Guyot, a post-master, at Versailles, who suffered from deafness. He introduced into the eustachian tube through the mouth. Our countryman, Cleland, soon after performed the operation through the nose, and used for this purpose flexible catheters. But the catheter now most commonly in use, and which was recommended by Laissy and Itard, and used by Kramer, Pilcher, and other surgeons of eminence, is an inflexible silver catheter, about six inches long, and of a calibre varying from the size of a crow-quill to that of a large goose-quill. The extremity is well rounded, and it should have a curve at about five lines from the further end, which should correspond with the lateral situation of the mouth of the eustachian tube. The catheters should be graduated with inches, which will be found useful in their repeated introduction.

In passing the catheter, the instrument should be warmed and oiled, and passed along the floor of the nostril with the convexity upwards and the concavity downwards. It should then be gently turned just before it reaches the pharynx, so that the point shall be outwards and a little upwards, as the mouth of the eustachian tube is above the level of the floor of the nose. The operator will readily feel when the catheter slips into the orifice of the canal.

It is a pity our author did not give more minute directions in reference to the performance of an operation attended with difficulty, and from the proximity of the catheter to the carotid artery and the delicate nature of the ear, certainly with no little danger. We have no doubt that the operation of passing the instrument into the orifice of the canal may be safely performed, and this will allow of the introduction of injections, air, vapors, &c., but to dilate strictures of the tube, or remove clots of blood from the tympanum, we should think its use of doubtful propriety.

Many of the cases of obstruction of the eustachean tubes, for which perforation of the membrana tympani was recommended by Sir Astley Cooper, may be relieved by means of the catheter, through which air may be forced into the tympanum. But little is said, as little is known of the diseases of the internal ear.

Chapter second opens on cerumenous concretions in the auditory canal. These are easily detected by looking into the ear. A small piece of looking glass, to throw the rays of the sun into the auditory canal, facilitates examinations very much. The treatment consists in syringing with warm water, at intervals of a few hours, until the mass is removed, and the use of sweet oil to keep the secretions soft. If any inflammatory symptoms attend it, counter irritation should be applied anterior to the ear. The following shows how carelessly even great men sometimes prescribe for the ear:—

Mr. Pilcher has related the case of a gentleman who had been afflicted with deafness of one ear for sixteen years, and had consulted Sir A. Cooper, Dr. Armstrong, and other eminent men, who had ordered blisters, and various other remedies, without any effect. He was at last recommended to undergo a course of warm-bathing for his general health, and one day, when in the bath, he heard a loud report, and fancied some one had shot at him, and on looking round, he found something floating upon the water about the size of a pea, which, upon examination, proved to be a lump of hardened cerumen. From that time he heard distinctly.

The section on herpetic ulcerations of the auditory canal presents nothing worthy of note, save the recommendation of the black wash as an application to them, which we should think a good practice.

The section on foreign bodies in the ear gives some very good suggestions in reference to the danger of too rude an operation for their removal, which certainly should be effected with care of the integrity of the membrana tympani. The means of removing foreign bodies from the auditory canal are the forceps, the scoop, the hook and probe. The best instrument will be found in a pair of forceps, with thin narrow jaws, and so slightly curved, that when opened within the auditory canal, the backs and points of the jaws shall be in close contact with the walls of the passage, so that they may slip by to embrace the substance, be it what it may.

A case is reported, in which the use of instruments roughly, resulted in death.

The next section is on worms and insects in the ear. The presence of entozoa in the ear is certainly possible, but highly improbable. The larvæ of insects are much more likely to be found, as they sometimes are in considerable quantities. Some five or six years ago, an Irishman, who labored on the public works, and slept in a shanty, applied to us from "a roaring, like a cataract," in his ear. Upon examination, it was found to contain maggots, large and plump, rolling and tumbling over each other, as is their manner. With a small pair of forceps, we removed between thirty and forty at a sitting. Our author recommends that they be driven from the bottom of the ear by oil of almonds, and then grasped with the forceps. Syringing and cleanliness of the ear are necessary as after treatment.

The last section in this chapter treats of polypi and excresences of the ear. Fungous from an ulcerated condition of the walls of the canal is occasionally met with. The knife and caustic are the remedies.

Deafness is sometimes produced by the meatus being closed by a polypus. Polypi arise from the lining of the auditory canal, or the membrana tympani, and are attached by a foot stalk. The treatment is removal by the knife or ligature.

The chapter on nervous diseases has a section on erithitic and on torpid nervous deafness. The first is defined as consisting in augmented sensibility; the second in diminished irritability. The first is attended by tinnitus aurium, which increases with the progress of the deafness. It is absent in the latter.

In the first case, constitutional treatment is recommended to relieve every symptom of disease that occurs, and tonics, a change of air, and counter irritants behind the ear, are the remedies.

In torpid nervous deafness, there is a decidedly hereditary tendency; according to Kramer, one third of the cases being traceable to this cause.

The symptoms are dryness and insensibility of the external ear and auditory canal, whiteness of the membrana tympani, &c.

Our author relies much upon injections through the eustachian tube, and the introduction of etherial vapors for its cure.

The chapter on the treatment of the deaf and dumb contains some valuable statistics. Scarlet fever seems to be first in frequency as a cause. The following gives the author's experience, and his recommendations in practice:—

The author has lately had an opportunity of examining several deaf-mutes, whose hearing was lost after birth; and in most of these cases the eustachian tube was either partially or totally obstructed, and an obviously inflammatory state of the throat existed. In one case, where the eustachian tube was free, the membrana tympani was thickened and opaque. In another, there was ulceration of the membrana tympani, with otorrhœa. In two cases there appeared to be no other disease than a narrowing or partial obstruction of the eustachian tube; and these individuals could distinguish and imitate sounds. How far these cases might be relieved, at this distance of time, by dilating the eustachian tube, and washing out the tympanum, is worthy of attention.

The treatmeut of these cases is often abandoned as hopeless. The author, however, has seen one case where hearing was restored by dilating the eustachian tube, and the application of the air-douche. In those cases where there is a deposit in the tympanal cavity in addition to the obstruction of the eustachian tube, it should be washed away by injecting water into the tympanum through the tube. The tympanum should afterwards have thrown into it an injection, composed of a small quantity of ether with water. In most of the cases examined by the author, there was an enlargement of the tonsils, with an inflamed state of the throat. In such cases, the throat and tonsils should be scarified, and where the tonsils are very much enlarged, they may be removed with a pair of scissors.

Children that are born deaf should have the eustachian tube explored as soon as their age will admit of it; and such children as have become deaf from disease, should be treated as early as possible.

We have thus given a synopsis of the work. It will be found useful, though much less extensive than one embracing the same range of subjects might profitably be; and embracing fewer subjects than might be advantageously comprised in a treatise on diseases of the ear.

An accurate and clear description of the anatomy and physiology of the internal ear would be an important prefix to such a work from the fact first mentioned, that too little attention has been paid the organ to for it to be sufficiently understood. E.

